# Devices for Ambulatory Monitoring of Sleep-Associated Disorders in Children with Neurological Diseases

**DOI:** 10.3390/children5010003

**Published:** 2017-12-25

**Authors:** Adriana Ulate-Campos, Melissa Tsuboyama, Tobias Loddenkemper

**Affiliations:** 1 Department of Neurology, National Children’s Hospital Dr. Carlos Saenz Herrera, 10103 San José, Costa Rica; adrianaulate@hotmail.com; 2 Division of Epilepsy and Clinical Neurophysiology, Department of Neurology, Boston Children’s Hospital, Harvard Medical School, Boston, MA 02115, USA; melissa.tsuboyama@childrens.harvard.edu

**Keywords:** sleep monitoring, sleep devices, seizure detecting devices, SUDEP, pediatric sleep obstructive apnea, parasomnias, actigraphy, ambulatory polysomnography

## Abstract

Good sleep quality is essential for a child’s wellbeing. Early sleep problems have been linked to the later development of emotional and behavioral disorders and can negatively impact the quality of life of the child and his or her family. Sleep-associated conditions are frequent in the pediatric population, and even more so in children with neurological problems. Monitoring devices can help to better characterize sleep efficiency and sleep quality. They can also be helpful to better characterize paroxysmal nocturnal events and differentiate between nocturnal seizures, parasomnias, and obstructive sleep apnea, each of which has a different management. Overnight ambulatory detection devices allow for a tolerable, low cost, objective assessment of sleep quality in the patient’s natural environment. They can also be used as a notification system to allow for rapid recognition and prompt intervention of events like seizures. Optimal monitoring devices will be patient- and diagnosis-specific, but may include a combination of modalities such as ambulatory electroencephalograms, actigraphy, and pulse oximetry. We will summarize the current literature on ambulatory sleep devices for detecting sleep disorders in children with neurological diseases.

## 1. Introduction

Good sleep quality is essential for a child’s development, growth, memory, and ability to learn [1]. Sleep plays a role in normal synaptic development, neural plasticity, brain maturation, and memory consolidation during early development [2,3,4]. Additionally, early sleep problems have been linked to the later development of emotional and behavioral problems in animal models and in several childhood cohorts [5,6,7,8,9]. Short sleep duration and frequent nocturnal awakenings at 18 months of age may predict behavioral and emotional problems at five years of age [10].

Sleep-associated disorders affect approximately 25% of children. They are more frequent in children with neurological problems, and have been shown to affect the quality of life (QOL) of patients and their families [5,11,12,13,14,15,16,17]. While parents and caregivers can use sleep diaries to monitor their child’s sleep habits, some details are often unaccounted for. Therefore, a monitoring device such as an actigraphy may help to better characterize sleep in patients at risk by tracking sleep onset latency, total sleep time, wake after sleep onset, sleep efficiency, and (tentatively) other events occurring during sleep and their frequency, allowing for more accurate data collection [18,19,20,21].

While a variety of pediatric sleep disorders exist, some of the conditions that require early diagnosis and treatment include nocturnal seizures, parasomnias, and obstructive sleep apnea [12,22,23]. Overnight detection devices may permit a more objective assessment of sleep quality and characterization of events, as well as earlier recognition and subsequent intervention in response to events such as obstructive sleep apnea and seizures. A variety of detection modalities are now available, including ambulatory electroencephalograms (AEEGs), electrocardiograms (EKGs), surface electromyography (sEMG), electrodermal activity (EDA) monitoring, actigraphy, video detection systems, mattress sensors, multimodal devices, nocturnal oximetry, and ambulatory polysomnography (PSG) that may be used as a single detection device, or in combination to further characterize sleep quality and paroxysmal nocturnal events [24,25,26,27,28,29,30,31,32]. Here, we will summarize the current literature regarding ambulatory sleep devices for monitoring sleep disorders in children with neurological diseases.

## 2. Monitoring Sleep Quality

Several neurological and neurodevelopmental syndromes present with sleep problems, including trisomy 21, Smith–Magenis syndrome, Rett syndrome, fragile-X syndrome, Angelman syndrome, and Prader–Willi syndrome, as well as attention deficit hyperactivity disorder (ADHD), cerebral palsy, tuberous sclerosis, mucopolysaccharidoses, Niemann–Pick disease type C, and autism spectrum disorders (ASD), among others [14,33,34,35,36,37]. The most common complaints in these syndromes involve difficulties with sleep initiation, excessively fragmented sleep, frequent awakenings, sleep-disordered breathing, inverted circadian rhythm, problematic nighttime behaviors, and daytime sleepiness [33,35,37]. Sleep problems have been reported in up to 75–80% of children with ASD, almost three times the prevalence in the general population [15,17,38,39], and include problems with initiating and maintaining sleep, as well as anxiety-associated sleep behaviors [40]. Ambulatory tools used for monitoring sleep quality include sleep diaries and actigraphy. Ambulatory PSG may also be helpful and details will be provided in the obstructive sleep apnea section.

### 2.1. Sleep Diaries

Sleep diaries are often used to track the quality of sleep and response to interventions [14,33,34,35,36,37,41,42]. However, parents of normally developing children and adolescents may not be aware of sleep onset latency and night awakenings, or may report an idealized quality and quantity of sleep. 

Children are more accurate in reporting their sleep habits and quality of sleep than parents, but in patients with severe neurodevelopmental disorders this may not be an alternative [43] There is a higher parental report of sleep problems and discrepancies with self-reports in children with ADHD, emphasizing the need for more objective measures [44,45,46]. Therefore, sleep diaries currently remain in use as a screening tool, but are being supplemented with wearable data obtained through, for example, actigraphy.

### 2.2. Actigraphy

Polysomnography remains the gold standard for assessing sleep quality, but the high cost and limited ability to provide information about one night of sleep outside of the child’s habitual home environment limits its practicality. For this reason, actigraphy has been evaluated as an alternative or supplementary option. Actigraphy is a noninvasive, cost-effective monitoring method that assesses sleep quality through an accelerometer that analyzes movements in different axes. From this data, it is able to provide measurements of total sleep time, avoiding the recall bias related to sleep diaries [47,48]. A small wrist- or ankle-worn device houses the accelerometer. There have been several studies that have validated its use in the pediatric population, with the notable advantages of low cost, with reasonable tolerability over long periods of time, and functionality in terms of its use in the child’s habitual sleep environment at home without major disruptions or distractions [19,20,49,50]. Actigraphy in children with ADHD is well tolerated, and has revealed higher motor activity, altered sleep efficiency, and prolonged sleep onset latency compared to normally developing children. Of note, sleep onset latency refers to the time it takes to transition from full wakefulness to sleep. This information obtained through actigraphy is comparable to that reported through PSG [18,46,51]. In one study, actigraphy was tolerated in 62 out of 69 children with ASD; only 7 patients refused to wear the actigraphs or removed them [40]. When compared to overnight home PSG, actigraphy has a sensitivity of 88% but specificity of 46% in children aged 5–12 years who were born prematurely [19]. In these sleep studies, sensitivity refers to the ability to detect true sleep and specificity to the ability to detect true wake [19]. Another study in 45 children ranging from 1 to 12 years old showed similar results, with a sensitivity of 90.1–97.7% and a specificity of 39.4–68.9% [49]. Studies assessing the use of actigraphy in patients with neurodevelopmental disorders, however, are scarce [20,49,50]. One study found that in children with ASD and developmental delay, the sensitivity of actigraphy was 97% and specificity was 24% [48]. The low specificity of actigraphy illustrates the main limitation of the device: its inability to distinguish movements during sleep from those of nocturnal awakenings [47,48,49].

## 3. Parasomnias and Nocturnal Paroxysmal Events

The most common paroxysmal nocturnal events in children with neurological disorders are parasomnias, and they form part of the differential diagnosis when ruling out epilepsy [39]. In one study, 29% of patients diagnosed with nocturnal frontal lobe epilepsy by polysomnography were originally referred for parasomnias [52]. Inpatient continuous video-electroencephalogram (EEG) monitoring is considered the gold standard for the diagnosis of epilepsy. However, given the high cost associated with the hospitalization required for this test, similar, more cost-effective and practical screening tests may also be performed in the ambulatory setting, i.e., ambulatory PSG or AEEG [53,54].

### Ambulatory Electroencephalogram

Ambulatory electroencephalogram consists of continuous monitoring for 24 h or more in the outpatient setting. When the suspected event, parasomnia, non-epileptic event, or seizure, occurs multiple times per week, AEEG may provide a reasonable likelihood of recording events during a 24–48 h recording period [55,56]. In a prospective study of 64 children ranging from 0 to 17 years of age, AEEG aided in the diagnostic evaluation of 73% of the patients and helped to differentiate seizures from non-epileptic events in 61% [53]. Addition of simultaneous video monitoring to view the semiology of the events facilitates interpretation of the electrographic recording, and ultimately the ability to differentiate between seizures and non-epileptic events. In children with neurological disorders, recording in the child’s home environment may be preferred since it often results in better tolerance of the procedure, and may permit recording of the event. Although AEEG devices eliminate the need for hospitalization, patients may find these devices cumbersome because they involve leads placement on the head, and the leads are attached to a set of wires and a recording device. Patients often have difficulties tolerating these leads for more than a few days, and the quality of the EEG recording also tends to decline successively, unless families or technicians repair electrodes that may lose contact during recordings. Counseling families and training caregivers on how to maintain optimal EEG lead integrity for as long as possible is also paramount to obtaining technically satisfactory recordings [57]. Some of the disadvantages of this device include the inability for a medical provider to evaluate the child during an event, the lack of video in most cases, and the inability to readjust, reposition, or repair problematic electrodes during the recording [53]. Ultimately, the information AEEG provides often serves as an adequate screening step and compromise between obtaining a routine EEG that offers only limited information, and obtaining an inpatient continuous video-EEG study that may be more taxing on the patient and family. Thus, there is ongoing research to develop wireless EEG modalities that may be less cumbersome to handle [58,59,60,61,62,63]. One example, among many others, is a small two-electrode ambulatory EEG monitoring patch that is able to track and record EEG signals for up to seven days [64].

## 4. Sleep-Related Epilepsy

Epilepsy has an incidence of approximately 41–187/100,000 per year in children, and 20–30% of these patients will continue to have more than one seizure per month despite optimal medical management [65,66,67,68,69]. Patients with active epilepsy have a higher mortality than seizure-free patients with epilepsy [70,71,72,73,74]. There is an increased risk of sudden unexplained death in epilepsy (SUDEP) in patients with generalized tonic–clonic seizures (GTCS), while unsupervised in bed at night [75,76]. Both seizures and interictal discharges can be activated or potentiated in non-rapid eye movements (NREM) sleep in selected epilepsy conditions [77,78,79,80]. Several devices have been evaluated for the detection of nocturnal seizures. Seizure detection is more accurate when it combines more than one modality; multimodal systems have shown increased sensitivity and lower false detection ratios [81,82,83,84,85,86]. Modalities in addition to EEGs that may improve ambulatory detection rates of nocturnal seizures include: EKG, sEMG, EDA, actigraphy, video detection systems, and mattress sensors. Devices may provide accurate seizure quantification for selected seizures, tentatively permitting the sooner application of a rescue medication through earlier detection, or facilitating chrono-therapy regimens permitting treatment at times of greater seizure susceptibility based on the time pattern of detected seizures [86,87].

### 4.1. Ambulatory Electroencephalogram

Video-electroencephalogram remains the gold standard for the diagnosis of epilepsy. Studies using algorithms for automatic EEG detection of seizures have revealed reasonable sensitivities for seizure detection [88,89,90]. Deployment of similar algorithms within an ambulatory EEG system could be used to detect nocturnal seizures at home. More information on AEEG is also provided in Section 3. Furthermore, a closed loop system could be developed in which the seizure is automatically detected by the EEG information by onboard processing and algorithms. Such a system would permit alerts, shortening the time to emergency interventions and treatment.

### 4.2. Electrocardiogram

Seizures can be associated with ictal tachycardia, tentatively making EKG a helpful ancillary detection modality. Cardiac arrhythmias and patterns such as T-wave alternans may also be associated with SUDEP, further supporting the use of EKG to detect seizures [91,92]. An EKG can be recorded from a single channel and has a higher signal-to-noise ratio than EEG [93]. Multimodal combination devices now include both an ultra-low power EKG sensor readout, and accelerometer for ambulatory monitoring of seizures. Other options utilize cardiac-based activated vagus nerve stimulation as part of a closed-loop system to detect seizures by delivering a stimulus when the heart rate is above a certain threshold; these may have an effect on seizure control and improvement in quality of life [94].

### 4.3. Surface Electromyography

Surface electromyography can also be used to detect the motor component of seizures [95]. Surface electromyography has good sensitivity for selected seizure types (ranging from 53 to 95% for GTCS and tonic seizures), particularly when placed over the deltoid, biceps, and triceps muscles [30,96,97]. However, sEMG sensors may cause discomfort when strongly affixed to the skin and have the potential for detachment [97].

### 4.4. Electrodermal Activity

Electrodermal activity detects changes in sweat excretion and is thought to largely reflect activity of the sympathetic nervous system. Since many seizure types involve increased activation of the sympathetic nervous system, this transient increase in EDA serves as another parameter that may help detect nocturnal seizures, in particular generalized tonic–clonic seizures [31,98]. Electrodermal activity measurements taken from the ventral side of the distal forearm have been tolerated, even when applied for long periods of time [31]. However, EDA recordings are susceptible to pressure and motion artifacts. Studies on continuous ambulatory EDA monitoring are needed to provide information on how to optimize this modality both in the general population and in children with neurological diseases, as well as in specific seizure types beyond generalized tonic–clonic seizures.

### 4.5. Actigraphy

Actigraphy has been used to identify motor seizures by detecting changes in velocity and direction of movement [32,99,100,101]. Although differentiating seizures from repetitive movements can present a challenge, actigraphy has demonstrated good accuracy for detecting nocturnal seizures, with one study reporting detection of 78.5% of the seizures reported by parents [102]. Most patients and families also found such a device user-friendly [32,99,101,103]. The main limitation of this device is that its use is restricted to the detection of seizures with motor components. Additionally, if there is an obstacle to free limb movement during a seizure, the seizure may not be detected [32].

### 4.6. Mattress Sensor Systems

Mattress sensor systems consist of a sensor placed under the patient’s mattress that is connected to a monitor with a detection algorithm. The device alerts caregivers when the sensor detects a stimulus above a set threshold or pattern [26,27,28]. One mattress sensor system had a sensitivity of 62.5% and a specificity of 90%, while another had a sensitivity of 85% for detection of GTCS during sleep [26,28,104]. The disadvantages of mattress sensors are that they: (1) are best for detection of seizures with rhythmic movements; (2) often have a weight restriction; and (3) often have lower sensitivity compared to other devices [27,28]. Individual calibration for different movements during sleep, and testing over a couple of nights in a home setting are recommended and may permit individually improved results [26].

### 4.7. Video and Sound Detection System

A promising and popular seizure detection modality is related to video detection of seizures. They are divided into two categories: marker-based or marker-free [25]. Marker-free systems only detect seizures with a motor component and are limited to the area covered by video [25]. Marker-based systems place reference video fiducials on the head, trunk, or extremities to assess finer movements detected with infrared light. Because of the additional equipment required for marker-based systems, these may be uncomfortable, and the additional sensors may be disconnected or out of the detection area of the camera [105,106]. Video detection systems are best for recognition of seizures with large amplitude movements. Video detection may also include recording of sound, and acoustic baby monitoring systems are often also used in seizure patients, to permit detection of seizure-specific noises or vocalizations.

### 4.8. Multimodal Devices

Multimodal systems are proposed as alternatives to video-EEG monitoring because they combine two or more modalities, resulting in higher sensitivities and lower false detection ratios of many seizure types, while allowing for home monitoring [85]. For example, the combination of EDA and actigraphy improves detection of seizures with a motor component and autonomic involvement. A system with actigraphy and EDA placed on patients with GTCS during awake and sleep states yielded 94% sensitivity for seizure detection and one false alarm per 24 h [107]. The multi-modal intelligent seizure acquisition (MISA) system includes sEMG, magnetometers, actigraphy, and gyroscopes which allow for the assessment and characterization of full body movements [81]. The MISA system was first tested on individuals who simulated seizures. The person specific system detected all seizures, with only one false positive in a four hour time frame [83]. Some subjects found the system uncomfortable, and therefore smaller and fewer electrodes may likely be used in the next prototype [83,84].

Overall, EEG, actigraphy, sEMG, and video and sound detection, as well as EDA, are the modalities that have been most frequently used to date, with reasonable sensitivity for generalized tonic–clonic seizures in many cases [86]. There is abundant ongoing research in this field which hopefully will introduce more sensitive multimodal devices that can be tailored to individual characteristics, neurological conditions and seizure types.

## 5. Obstructive Sleep Apnea

Pediatric obstructive sleep apnea (OSA) is defined as a breathing disorder during sleep characterized by prolonged partial or complete upper airway obstruction that disrupts both normal ventilation during sleep and normal sleep patterns [108,109]. Its prevalence in the pediatric population is up to 5.7% [109]. A meta-analysis of 350 studies found that OSA in children was linked to deficits in cognition and neurophysiological function [109]. Deficits in executive functions and memory were the most common cognitive problems, and hyperactivity was the most common behavior abnormality [109,110,111,112]. Nocturnal, in-laboratory PSG is the gold standard for diagnosing OSA, but some lower cost alternatives including sleep questionnaires, nocturnal oximetry, and home-based PSG have been evaluated [109,113]. These options may be preferred in some patients as a screening tool, especially in children with neurodevelopmental disorders as they are able to remain in a familiar environment.

### 5.1. Sleep Questionnaires for Obstructive Sleep Apnea

Sleep questionnaires are offered as a simple, low cost alternative to detect pediatric OSA. The Pediatric Sleep Questionnaire is a 22-item sleep-related breathing disorder tool that centers on behavioral problems, excessive daytime sleepiness, and snoring. However, this questionnaire had a sensitivity of 78% and specificity of 72%, limiting its ability to be used as a surrogate for PSG [114]. Instead, sleep questionnaires can be used as a screening tool to identify patients who require additional diagnostic testing [109].

### 5.2. Home Oximetry

Home oximetry has the advantage of being of lower cost and more readily available than PSGs, which often have long wait-lists. One study found the use of home oximetry in healthy children older than 4 years old to have a sensitivity of 67% and a specificity of 60% [115]. Other studies have found the use of oximeters to have higher specificity but lower sensitivity in diagnosing pediatric OSA [24]. One study in children with a median age of 4 years found that 78% of recordings were either normal or non-conclusive, requiring a subsequent PSG [116]. A major contributor to the poor sensitivity and specificity of this device is oximetry movement artifact that results from frequent movements of children at night. Phone oximetry is yet another portable, easy to use screening tool with a sensitivity of 88% and a specificity of 83% in diagnosing OSA [117]. Ultimately, home oximetry may serve as another screening tool to be used in children with sleep disordered breathing to identify those who require a PSG or to estimate the severity of pre-existing OSA [116,118]. Alternatively, it can be used in combination with other devices such as an accelerometer and EKG for home monitoring of sleep-disordered breathing. Validation trials to identify the best combination of devices are still needed [119]. There is no single statistic on the number of children with neurologic disorders with OSA who do not suffer from desaturations, but more widespread use of portable devices may provide such data soon [120].

### 5.3. Ambulatory Polysomnography

Ambulatory PSG refers to unattended sleep studies conducted at home. This testing approach can produce good quality recordings, is well-tolerated, and is less disruptive to the patient’s schedule [109,121,122]. It is also less costly than an in-laboratory PSG. Ambulatory PSG was performed in a large cohort of 157 children from 5–12 years of age in the TuCASA study which demonstrated that ambulatory PSG recordings could be of high quality and not disruptive to sleep [122]. Five of these children also had in-laboratory PSG, and the results were comparable to those on the ambulatory PSG. Ninety-one percent of recordings were deemed of acceptable quality after the initial attempt. Similar findings were obtained by another study that assessed the feasibility of performing ambulatory PSG in children by performing this procedure in 201 ex-preterm children 5–12 years of age, including children with cerebral palsy or developmental delays. Ninety-one percent of cases had satisfactory recordings, and nearly all children tolerated the procedure well. The median parental satisfaction on a Likert scale was 1 (range 1–5, with 1 being best), and a median rating of 0 on the pediatric Likert scale (range 0–5, with 0 being the best) [121]. None of the participating centers had prior experience with ambulatory PSG and still achieved very good quality recordings that further improved in quality over 4 years of testing. In this particular study, technicians went to the home to set up the equipment including a full PSG montage, which likely contributed to the good quality of recordings. Access to these resources may not be widely available [121]. As feasibility has been shown, additional studies are now needed to validate ambulatory PSG as a diagnostic tool for OSA.

Children with ASD or anxiety are more likely to have difficulties tolerating most procedures, including ambulatory PSG, because of the unexpected sensory stimulus the equipment may produce or because of the novelty of the procedure. A desensitization protocol implemented in children with ASD and developmental delay was found to be successful in 86% of children with ASD and in 87% of those with developmental delay [123]. More widespread use of this desensitization protocol may be helpful to increase the likelihood of tolerability of this procedure in children, particularly in those with neurodevelopmental conditions [123].

## 6. Challenges and Future Directions

One of the main challenges in the development of an ambulatory device suitable for monitoring sleep in children with neurological disorders is the great number and variability of neurological disorders. However, once devices become more widely used, additional data on more rare conditions will become available. Another challenge may be increased sensitivity to devices in skin contact, as seen in allergic conditions or with autistic spectrum disorders, and many children with neurologic comorbidities may be hyperactive or hypersensitive to tactile stimuli. This highlights the importance that the device should be wireless, miniaturized, lightweight, and with minimal electrodes/skin contact. The ideal monitoring device should also be safe, comfortable, easy to use, and cause minimal disruption for patients, their caregivers, and—as applicable—for the medical staff using the device. Another important challenge is the limited number of studies regarding ambulatory sleep monitoring, and the great diversity in terms of research methodology among them. Nonetheless, all of the available information indicates that the optimal device for monitoring the quality of sleep should include a combination of sensor modalities, since multimodal devices are more sensitive than the isolated individual sensors. The optimal combination of modalities may need to be individualized based on the clinical patient presentation and the clinical question at hand. Devices may present with onboard processing and algorithms to detect an event, and could trigger an alarm that alerts caregivers and permits proper management. Finally, collaboration between patients, caregivers, physicians, and researchers may allow the development of devices that fulfill most of the requirements for it to be useful. This may also allow uniformity of research and improve the quality of the information provided.

## 7. Conclusions

The optimal device for ambulatory monitoring sleep in children with neurological conditions should be patient- and disease-specific, comfortable, and should involve a combination of detection modalities. We provided a table as a summary and quick reference guide to devices and indications for their use (Table 1). These devices are currently under development, and are urgently needed to respond to tentative emergencies quickly, and to minimize long-term consequences of chronic conditions.

## Figures and Tables

**Table 1:** Suggestions for monitoring of sleep and related indications with portable devices (choices are not exclusive, and may vary based on available resources and physician preference).

	Device/Instrument											
Indication	Actigraphy	Ambulatory EEG	Ambulatory PSG	EDA	EKG	Home Oximetry	Mattress Sensor Systems	Sleep Diary	Sleep Questionnaire	sEMG	Video Systems	Multimodal Devices
Sleep quality	X		X					X	X			X
Parasomnias		X									X	X
Nocturnal epilepsy	X	X		X	X	X	X			X	X	X
Obstructive sleep apnea			X			X			X			X

EEG: electroencephalogram; EDA: electrodermal activity; EKG: electrocardiogram; PSG: polysomnography; sEMG: surface electromyography.
